# Estimation of the Basic Reproduction Number and Vaccination Coverage of Influenza in the United States (2017-18)

**Published:** 2018-09-22

**Authors:** Roya Nikbakht, Mohammad Reza Baneshi, Abbas Bahrampour

**Affiliations:** ^1^ Department of Biostatistics and Epidemiology, Modeling in Health Research Center, Faculty of Health, Institute for Futures Studies in Health, Kerman University of Medical Sciences, Kerman, Iran

**Keywords:** Basic reproduction number, Epidemic threshold parameters, Vaccination coverage, Influenza

## Abstract

**Background:** Determining the epidemic threshold parameter helps health providers calculate the coverage while guiding them in planning the process of vaccination strategy. Since the trend and mechanism of influenza is very similar in different countries, we planned a study with the mentioned goal by using data of US from 2017 to 2018.

**Study design:** A secondary study.

**Methods:** R_0_ and corresponding vaccination coverage are estimated using the national and state-level data of the US from the 40^th^ in 2017 to the 5^th^ week in 2018. Four methods maximum likelihood (ML), exponential growth (EG), time-dependent reproduction numbers (TD), and sequential Bayesian (SB) are used to calculate minimum vaccination coverage. The gamma distribution is considered as the distribution and the generation of time.

**Results:** The peak of epidemy in most states has occurred in the 15^th^ week after the beginning of the epidemics. The generation time obey the Gamma distribution with mean and standard deviation of 3.6 and 1.6, respectively, was utilized for the generation time. The R_0_ (vaccination coverage) equaled 1.94 (48.4%), 1.80 (44.4%), 3.06 (67.3%), and 2.11 (52.6%) for EG, ML, SB, and TD methods at the national level, respectively.

**Conclusion:** The R_0_ estimations were in the range of 1.8-3.06, indicating that an epidemic has occurred in the US (R_0_>1). Thus, it is required to vaccinate at least 44.4% to 67.3% to prevent the next epidemics of influenza. The findings of this study assist futures studies to apply disease control by vaccination strategies in order to prevent a national disaster.

## Introduction

Clinical research and studies of communicable (infectious) diseases follow different purposes such as determining the trend, epidemic threshold parameters, and vaccination coverage. The epidemic threshold parameter, R_0_, plays a key role in the diagnosis of suggested control strategies in order to apply interventions or vaccination preventative strategies. In biostatistics and epidemiology, epidemic threshold parameter (whose special form is known as the basic reproduction number or reproductive ratio) defined as the mean number of secondary cases infected by initial cases in a fully susceptiblecommunity^1-3^. The basic reproduction number is generally compared with unity to assess the spread of infectious diseases to the population. An R_0_ greater than unity (R_0_>1) means an epidemic has occurred and each infected individual generates more than one new case^4^. In addition, the epidemic likely fades out when the basic reproduction number becomes less than unity (R_0_<1) and the R_0_ equals1, leading to an epidemic^1-2,5^.

Several approaches have been suggested to estimate R_0_. These include maximum likelihood (ML), exponential growth rate (EG), estimation of time-dependent reproduction numbers (TD), attack rate, gamma-distributed generation time, the final size of epidemic, and Richard model^6-14^. The type of approach depends on the type of data which is being studied (the type of household or daily incidence data). In each method, the basic reproduction numbers are reviewed to assess the intensity of interventions and vaccination strategies that can estimate the vaccination coverage of an infectious disease. Therefore, vaccination strategies are introduced in order to reduce and prevent the risk of transmission of infectious diseases in the target community. In addition, R_0_ and vaccination coverage have a direct impact on each other, which means that any variation in R_0_leads to a corresponding variation in vaccination coverage and vice versa. Therefore, a larger number of people should be vaccinated in a susceptible population where the estimation of basic reproduction number is a larger number.

The estimation of the basic reproduction number (R_0_) has been addressed in various infectious diseases, including influenza, HIV, SARS, smallpox, malaria, yellow fever, measles, and Ebola^15-22^. In particular, influenza is a leading cause of mortality, with a considerable number of annual deaths in the world ^23^. Several influenza epidemics have occurred worldwide from 2009 to 2017 during which a substantial number of people died annually^24^. For example, the number of deaths caused by “Asian flu” and “Hong Kong flu” is estimated at at1 to 4 million^25^. On the other hand, the annual deaths attributed to influenza are estimated at nearly 250,000 to 500,000 globally^26^. Moreover, United States flu (pH1N1) killed about 12469 persons in 2009^27^.

An epidemic has recently occurred in the US. Of course, the epidemic of influenza has been recorded in the US every year over the past 20 years, showing a yearly seasonal threat of the influenza epidemic^28^ (Table 1). A few of these epidemics are listed below:

**Table 1:** R_0_ estimation by different methods for USA data (2017-18)

**Variables**	**Method**			
	**Exponential** ** Growth (EG)**	**Maximum** **Likelihood (ML)**	**Sequential** **Bayesian (SB)**	**Time** **Dependent (TD)**
At the national level				
R0 (95% CI)	1.93 (1.93, 1.94)	1.80(1.78, 1.80)	3.05 (3.03, 3.08)	2.11 (2.10, 2.12)
Vaccination Coverage (%)	48.4	44.4	67.3	52.6
Among 52 states				
Var (95% CI)	0.04 (0.03, 0.07)	0.03 (0.02, 0.05)	0.02 (0.01, 0.03)	0.04 (0.03, 0.06)

In Philadelphia and New York (Sep 14 to Oct 17, 1918), the estimated R_0_ (95%CI) and generation time were 2.14 (1.88, 2.39) and 2.5 d, respectively. The formula used for estimating R_0_ was R_t_=∑_i_>0 IR_t_+_i_w_i_^29^. In the USA (1972-2002) reported the R_0_ (95% CI) of 1.30 (1.20, 1.40) with generation time of 5.5 d and formula R_0_=β/(γ+δ)^30^. Yang et al. estimated R_0_ of influenza using epidemiological surveys and mathematical modeling approaches in two periods of 2003-2004 and 2012-2013 are taken into account generation time of 4.2 and 4.8 respectively. The R_0_ (95% CI) in this study were estimated 2.04 (1.84, 2.21) and 1.97 (1.84, 2.21)^31^. The estimated R_0_ in the year 2009 (Mar 28- Mar 04) using likelihood-based method had a range 2.50 to 3.48 with corresponding 95% CI (1.80, 2.16) and (1.84, 2.13)^32^.

The R_0_s reported above have been obtained using different methods and generation times and are, therefore, impossible to be compared. Therefore, another aim of the present study was to calculate the R_0_ of influenza for a given set of data (USA data) considering the same distribution for generation time by different methods to be able to compare various approaches. The number of cases and generation time distribution is needed which is gamma distribution with mean and SD 3.6 and 1.6 respectively based on similar study.

In addition, determine the epidemic threshold, helps health providers calculate the coverage while guiding them in planning the process of vaccination strategy. Vaccination coverage is directly computed by R_0,_ called indirect effect/herd protection^33^. Indirect vaccination coverage is not only economic but also prevent epidemic which its effect exceed the direct effect^34^.

Since the trend of influenza is very similar in different years, we conducted this study to determine the epidemic threshold parameters and, consequently, the vaccination coverage in the US from 2017 to 2018.

## Methods

### Statistical Analysis

In the first step, the classic SIR (susceptible, infected/infectious, and removal) compartmental model used to describe the process of influenza was implemented to determine the protocol of the person transmission indifferent states.

Next, four methods were developed to estimate the R_0_ based on cumulative case count data in R statistical software (version 3.4.2) with R_0 _packages, including ML, EG, sequential Bayesian method (SB),and TD. All the methods were used in different papers for influenza data, so for this type of data all the methods could be used to estimate the R_0_.

It is necessary to have a distribution for the generation time in each method defined below. Moreover, a brief overview of each method is presented.

### Generation time

The length of time between infection in a primary infection and a secondary infection is defined as generation time or serial interval^35^.

**EG:** The R_0_ formula in the exponential growth rate method $is\mu_{t}=R(\sum_{i=1}^{t}N_{t-i}w_{i})\;where\;R=\frac{1}{M(-r)}.$ Here, "r", "M", and N_t_ demonstrate the growth rate of the infection population, the moment generating function of the generation time distribution, and cases over a consecutive time unit, respectively, and parameter "w" represents the generation time. In order to estimate the growth rate parameter, Poisson regression method is applied^7^.

**ML:** In this method, the distribution of secondary cases infected by primary cases assume Poisson with mean R. Suppose N_0_,N_1_,…, N_T_ represent cases over time and parameter w shows generation time. Then, the log-likelihood function based on Poisson distribution is as follows:

$$\begin{array}{l} LL(R)=\overset{T}{\underset{t=1}{\sum }}\frac{\exp{\left(-\mu_{t}\right)}^{N_{t}}}{N_{t}!}\\ \mu_{t}=R\overset{t}{\underset{i=1}{\sum }}N_{t-i}w_{i} \end{array}$$

The maximum of log-likelihood function gives the reproduction number (R)^36^.

**SB:** Suppose N(t+1) denotes incidence in time (t+1) for the SIR model where we have an approximate Poisson distribution with mean N(t)eγ(R-1) (ϒ shows the average generation time). A non-informative prior for R is used in the Bayesian framework. The posterior distribution of R in the previous day is applied as the prior distribution for R in the new day. The posterior distribution for R is as follows:

$$P(R|N_{0},...,N_{t+1})=\frac{P(N_{t+1}|R,N_{0},...,N_{t})P(N_{0},...,N_{t})}{P(N_{0},...,N_{t})}$$

The exponential distribution applies for generation time in this method ^11^.

**TD:** R_0_ can be estimated by TD with formula $R_{t}=\frac{1}{N}\sum \left\{t_{j}=t\right\}R_{j}\;where\;R_{j}=\sum_{i}p_{i}\;and\;p_{ij}=\frac{N_{i}w(t_{i}-t_{j})}{\sum_{i\neq k}N_{i}w(t_{i}-t_{k})}$. In this formula, P_ij_ demonstrates the probability of infection transmission form casei (in time t_i_) to case j (in time t_j_). R_t_ is the mean of all R_j_computed by all networks of observed cases^6^.

### Vaccination coverage

To compute the percent of vaccination coverage, we need to estimate R_0_. Therefore, the critical vaccination coverage, i.e. the proportion of people who receive vaccines, is obtained by the reproduction number using the following formula:

$$v=1-\frac{1}{R_{0}}$$

Vaccination coverage is also defined as the reduction in the probability of infection risk which is a value between 0 and 1^33^.

Vaccine efficacy for reducing transmission can be achieved by $\vartheta =\frac{R_{0}-1}{R_{0}-R_{1}}=\frac{1-1/R_{0}}{1-ab}$ that *a* and *b* represents susceptibility effect and infectivity effect respectively^37^.

### Data

The four above-mentioned models were fitted to the US 2017-18 pH1N1 data, applying FluView weekly report achieved from the Centers for Disease Control and Prevention (CDC) website ^38^. The data of Surveillance Network (ILINet) were implemented, reporting influenza cases in all 47 states, the District of Columbia, New Dakota, New York City, Puerto Rico, and the U.S. Virgin Islands for each age group in the 40^th^ week in 2017 to 5^th^ week in 2018.

For all states, we estimated the R_0_ based on four methods. Afterward, we calculated the variances of R_0_s for each method to check for variability among different states, and then hierarchical cluster analysis was applied as an explorative technique to specify the number of clusters in the K-means clustering method to cluster the states. Cluster analysis was performed in Minitab 17 statistical software (Minitab Inc., State College, PA) and IBM SPSS Statistics 22 (Chicago, IL, USA).

## Results

**Figure 1:**
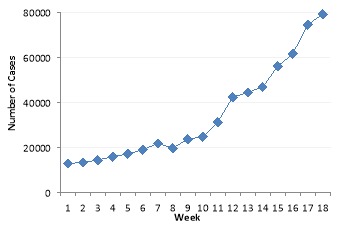
The incidence case count data of USA during 2017-18 (national-level)

The incidence data are presented on a weekly basis and all dates of USA data are based on week/year from the 40^th^ week in 2017 to the 5^th^ week in 2018. The peak of epidemy in most states has occurred in the 15^th^ week after start of the epidemics. The number of infected cases at the national level is provided in Figure 1.

**Figure 2:**
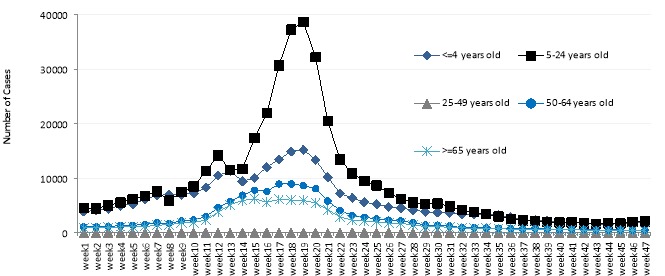
The incidence case count data of USA during 2017-18 (national-level) based on age group

The number of infected cases was plotted at the national level in five age groups. Maximum numbers of cases were in the age group of 5 to 24 yr and the peak incidence of influenza in this category occurred in the 19^th^ week (Figure 2).

The gamma distribution with the mean of 3.6 d and standard deviation of 1.6 d has been used as the distribution of the generation time ^1^. The results will be presented in three parts: national level, state level, and comparison of methods.

### National level

The national R_0_ and vaccination coverage are summarized in Table 1 based on four methods (ML, EG, SB, and TD) while assuming a wholly susceptible population before the start of the epidemy.

At the national level, the highest value of R_0_ was attributed to SB. Indeed, the estimated R_0_ at the national level by SB was quite different compared to other methods (3.057 95% CI: 3.037, 3.08). Moreover, the estimated R_0_s for EG, ML, and TD were 1.939 (1.937, 1.940), 1.80 (1.789, 1.802), and 2.111 (2.102, 2.120), respectively.

In addition, the estimates of vaccination coverage varied for the four methods, from 44.4% to 67.3%. The lowest and highest vaccination coverage values in this setting were associated with and SB methods, respectively (Table1).

### States Level

The computed R_0_s (95% CI) and vaccination coverage for all states by EG, ML, SB, and TD are summarized in Table 1 in the appendix. In general, the estimates of R_0_ at state level ranged from 1.55 to 2.79 for EG, 1.48 to 2.65 for ML, 1.62 to 2.46 for SB, and 1.67 to 2.73 for TD.

For each method, the variance of R_0_among states was calculated and the equality of variance with zero was tested by the chi-squared test (Table 1). The results for all methods indicated a variation in R_0_s among states because the reported confidence intervals did not include zero. Therefore, the states can be clustered using cluster analysis.

These 52 states were divided into two clusters based on hierarchical clustering. Next, the results were used for k-means clustering applying k=2. The first cluster included 12 states and the second one included 40 states. The overall results of the K-means cluster analysis are presented in Table 2.

**Table 2:** The R_0_s (95% CI) and vaccination coverages of two clusters for all methods

**Variables**	**Exponential Growth (EG)**	**Maximum Likelihood (ML)**	**Sequential Bayesian (SB)**	**Time dependent (TD)**
Cluster1				
R0 (95% CI)	2.08 (2.07, 2.08)	1.85 (1.85, 1.86)	2.07 (2.02, 2.12)	2.24 (2.20, 2.28)
Vaccination Coverage	0.52	0.46	0.52	0.55
Cluster 2				
R0 (95% CI)	1.81 (1.81, 1.81)	1.67 (1.67, 1.67)	2.97 (2.95, 3.01)	1.98 (1.97, 1.99)
Vaccination Coverage	0.45	0.40	0.66	0.49

**Cluster1:**Delaware, Idaho, Iowa, Kansas, Kentucky, Missouri, New Dakota, New Mexico, Rhode Island, Tennessee, Utah, Washington **Cluster2:** Alabama, Alaska, Arizona, Arkansas, California, Colorado, Connecticut, District of Colombia, Georgia, Hawaii, Illinois, Louisiana, Maine, Maryland, Massachusetts, Michigan, Minnesota, Mississippi, Montana, Nebraska, Nevada, New Carolina, New Hampshire, New Jersey, New York, New York City, Ohio, Oklahoma, Oregon, Pennsylvania, Puerto Rico, South Carolina, South Dakota, Texas, Vermont, Virginia, West Virginia, Virgin Islands, Wisconsin, Wyoming

In the first cluster, the lowest value of R_0_ for EG and ML is related to Idaho (EG: 1.97 95% CI (1.92, 2.03), ML: 1.48 95% CI(1.47, 1.50)). In addition, New Mexico had the minimum value of R_0_ for SB and TD (SB: 1.99 95% CI(1.90, 2.09), TD: 2.16 95% CI (2.08, 2.23)). Besides, the highest R_0_ was estimated for Delaware using EG, ML, andTD (EG: 2.7995% CI (2.69, 2.89)), ML (2.6595% CI (2.52, 2.79)), and TD (2.73 95% CI (2.12, 3.35)), and for New Dakota using SB (2.46 95% CI (1.84, 2.97)).

Overall, in the first cluster, the estimated R_0_ for EG, ML, SB, and TD equaled 2.082 95% CI(2.077, 2.088), 1.858 95% CI(1.850, 1.867), 2.077 95% CI(2.025, 2.127), and 2.244 95% CI (2.204, 2.283), respectively, where the minimum and maximum values of R_0_belonged to ML and TD, respectively. Except for ML, other methods yielded a vaccination coverage of more than 50%.

In the second cluster, the R_0_s calculated by four methods had the minimum value for Arkansas. New Hampshire had the largest R_0_ for EG and ML. Moreover, the R_0_ was maximum in Oklahoma for SB and TD. In total, the estimated R_0_ (95% CI) in the second cluster equaled 1.818 (1.817, 1.819) for EG, 1.676 (1.674, 1.678) for ML, 2.976 (2.956, 3.01) for SB, and 1.986 (1.978, 1.994) for TD. More than 40% vaccination coverage is required for the states in the second cluster.

Despite the low vaccination coverage percent determined in Arkansas, there were states where the vaccination coverage had a large calculated percent. Therefore, at the state level, the percentages of vaccination coverage calculated using EG, ML, SB, and TD were the lowest for Arkansas (EG: 35.5%, ML: 32.4%, SB: 38.3%, and TD: 40.1%). Furthermore, the highest vaccination coverages (EG:64.1%, ML:62.3%, and TD:63.4%) were associated with Delaware.

### Comparison of methods

Except for the national level, the R_0_ across all the states (in both clusters) had an approximately identical estimation for both SB and TD. In other words, the estimated R_0_ using SB were consistent with that calculated using TD. The R_0_s estimated by ML were slightly less than those estimated using EG. The variance (95% CI) of R_0_s among these four approaches equaled 0.32 (0.10, 4.51), indicating variability. Cluster analysis based on the mentioned methods resulted in two clusters. The first cluster included EG and ML, while the second cluster comprised SB and TD. This analysis confirmed the above findings.

## Discussion

The simple SIR compartmental model is used as the transition model, indicating that the estimation of R_0_using ML, EG, SB, and TD varied in different states due to the difference in the number of infectious cases during the outbreak. The variability of R_0_ depends on many factors, including location, estimation method, generation time, and pandemic wave,^39^. The virus and network size are also influential factors in influenza transmission^10^. The peak value of outbreak was the same in most states. Moreover, a sharp peak was observed in the incidence of H1N1 at the national level (Figure1).

We have found variation in the estimation of R_0_ using ML, EG, SB, and TD implemented by the "R_0_ package". A quantitative comparison of findings revealed that the estimations of R_0_ in SB are approximately close to those of TD. Besides, EG and ML yielded almost identical results, and cluster analysis based on the four methods confirmed this hypothesis.

The estimated epidemic threshold values based on three methods (EG, ML, and TD) in the first cluster were higher than those of the second cluster. Consequently, states in the first cluster have a higher risk of epidemic and require more vaccination coverage.

Generally, the R_0_associated with ILINet using four methods was greater than the one at the national level (winter of 2017) as well as state level (in both clusters), representing the epidemic of influenza. Therefore, it seems necessary to consider appropriate solutions to control, decrease, and prevent the epidemic or pandemic of influenza. An effective way to protect people from the attack rate of influenza is vaccination. Annual vaccination against seasonal influenza provides protection in high-risk groups (elderly people, ill persons, pregnant woman, and children) and can also reduce mortality rate, the incidence of disease, exacerbations, hospitalizations, and costs.

In determining vaccination coverage, R_0_ plays a key role because the estimation of vaccination coverage is affected by R_0_ (v=1-1/R_0_). In other words, the percentage of a community vaccinated against influenza can be represented in terms of R_0_. Vaccination coverage and R_0_directly impact each other, which means that, with an increase in R_0_, vaccination coverage increases, and vice versa. The present study also provides an estimate of vaccination coverage for both national and state levels, which is one of the strengths of this study.

The R_0_ of influenza for USA ranged between 1.3 and 3.1 from 1918 to 2013 using various methods ^35-36, 38-39^. In our study, similar values were obtained for R_0_. For example, at the national level, the R_0_wasestimated using four methods (ML, EG, SB, and TD) and their values were in the range of 1.8 to 3.06, indicating that an epidemic occurred in USA (R_0_>1).

Various studies have employed different methods and generation times to estimate the threshold of epidemics. Thus, it would be illogical to make such comparisons. In our study, the R_0_ of influenza for USA data considering the same distribution for the generation time in different methods so that various approaches can be compared, which is another strength of this study.

A weakness of this study was that, although cluster analysis determined similar methods, there was no exact criterion for determining the best method.

## Conclusion

The findings of our study can be used to improve policy-making, health care, and public health not only in the USA but also in other parts of the world. These results can be extended to other countries with similar epidemics. As the transmission mechanism is the same, the influenced parameters of the disease should be the same. In other words, the epidemic of influenza is similar in all countries so, in our country by at least 44.4% of vaccination can prevent the flu outbreak. Hence, awareness of the R_0_ of influenza as a highly infectious disease is helpful for futures studies to apply disease control through vaccination strategies in order to prevent a national disaster. Indirect vaccination coverage is not only economic but also prevent epidemic which its effect exceed the direct effect^34^.

Influenza would become re-epidemic. Therefore, a more comprehensive study is needed to deal with this dangerous virus.

## Acknowledgments

This research is part of Roya Nikbakht’s PhD dissertation.

## Conflict of interest statement

The authors declare no conflict of interest.

## Funding

There was no funding resource for this study.

## Highlights

- The R_0_ (vaccination coverage) equaled 1.94 (48.4%), 1.80 (44.4%), 3.06 (67.3%), and 2.11 (52.6%) for EG, ML, SB, and TD methods at the national level, respectively.
- The R_0_ estimations indicating that an epidemic has occurred in the US (R_0_>1).
- It is required to vaccinate at least 44.4% to prevent the next epidemics of influenza in the US.
- The estimated R_0_ using SB were consistent with those calculated using TD.
